# Physical Activity Behavior Change Driven by Engagement With an Incentive-Based App: Evaluating the Impact of Sweatcoin

**DOI:** 10.2196/12445

**Published:** 2019-07-08

**Authors:** Mark Elliott, Felicia Eck, Egor Khmelev, Anton Derlyatka, Oleg Fomenko

**Affiliations:** 1 Institute of Digital Healthcare WMG University of Warwick Coventry United Kingdom; 2 Sweatco Ltd London United Kingdom

**Keywords:** physical activity, incentives, rewards

## Abstract

**Background:**

Physical inactivity, now the fourth leading cause of death, is a primary element of noncommunicable diseases. Despite a great number of attempts, there is still a lack of effective approaches that can motivate sedentary populations to increase their levels of physical activity over a sustained period. Incentives for exercise can provide an immediate reward for increasing activity levels, but because of limited funding to provide rewards, previous programs using this approach have only shown short-term changes in behavior. Sweatcoin (Sweatco Ltd, UK) is an app-based platform that converts physical movement into virtual currency. The currency can be exchanged for goods and services on their marketplace, providing a continuous incentive to be active. This study investigates the physical activity behavior change observed in Sweatcoin users over a 6-month period of app usage.

**Objective:**

The aim of this study was to investigate the change in physical activity (measured using daily step count) of a sample of Sweatcoin users, the longevity of the change, and whether this change can be predicted by demographic and other lifestyle variables.

**Methods:**

Activity data from a sample of 5892 Sweatcoin users were used to analyze daily step count. Activity change was measured in terms of the percentage change in average daily step count for each month after registration, relative to that in the 3 months before using the app. Users were grouped according to having no or negative, moderate, or high activity change. A subset of users completed a questionnaire that allowed differences between groups in terms of activity and demographic status to be investigated using regression analyses.

**Results:**

Daily step count increased by 19% on average over the 6 months following registration (*P*<.001). Of the questionnaire respondents, 728 were valid responses. A multinomial logistic regression identified the key drivers of moderate and high activity behavior change relative to no or negative change based on the defined groupings. There was a clear impact of seasonality, with those registering for the app in winter (odds ratio [OR] 4.67; *P*=.001) and spring (OR 5.05; *P*=.001) being more likely to show high positive activity behavior change than those registering in summer. More striking were the results identifying those classified as overweight (measured through body mass index [BMI]; OR 1.83; *P*=.02) and less active (based on a self-reported scale of physical activity; OR 0.88; *P*=.048), being most likely to show high levels of physical activity change following registration with the app.

**Conclusions:**

The results highlight that an incentives-based app can induce significant physical activity behavior change, sustained over a 6-month period. Importantly, the results suggest that those typically lacking motivation to exercise (sedentary and high BMI) are most likely to be incentivized to increase their activity levels.

## Introduction

High levels of sedentary behavior increase the risk of cardiovascular disease, some types of cancer, and type 2 diabetes, particularly when combined with low levels of physical activity [1]. Europe has the highest levels of sedentary behavior, with nearly two-thirds of the population estimated to spend an average of 4 hours or more per day sitting [2]. Globally, the prevalence of insufficient physical activity was 27.5% in 2016 [3], with an estimated cost to health care systems of US $53.8 billion in 2013 [4]. The thresholds for improving the levels of physical activity for benefit are relatively low: regular bouts of walking have long been identified to be advantageous to many aspects of physical and mental health [5,6]. Yet, for sedentary individuals, there is often a lack of motivation to increase the levels of exercise, despite its value in terms of quality of life.

Adherence to physical activity programs has been found to be higher in individuals who exercise for enjoyment and social interaction rather than for fitness and appearance [7]. This can be aligned to the long- and short-term rewards leading to motivation levels: those focusing on longer-term rewards (fitness and appearance) are less likely to continue with a physical activity program than those focusing on the instant rewards of enjoyment and social interaction. This can be explained by systematic biases toward immediate reward in human behavior, known as present bias [8]. Therefore, providing immediate rewards to inactive individuals, in the form of incentives, could be a way of providing the necessary motivation to increase activity [9]. Evidence to date has shown this to be effective in a number of studies, albeit over short periods. Extending incentives-for-exercise programs beyond short-term trials is costly; however, because of the extrinsically motivated approach, there is a risk that individuals engaged with such short-term programs could regress soon after the incentive structure has been removed [10]. The lack of continuous engagement with program participants makes it more difficult to drive sustainable levels of activity behavior change, even if the program delivers behavior change in the short run. Therefore, a program of sustained goals and rewards is required that will continue to motivate sedentary populations to become more active. Wearable and smartphone technologies provide an opportunity for these programs to be implemented on a large scale, at relatively low cost. Adopting this approach, Sweatcoin is a concept and an app that converts the step count recorded on smartphones into a virtual currency. Hence, the users generate financial rewards through physical activity, which can be used to purchase products and services from an in-app marketplace. Sweatcoin has had more than 20 million users installing the app in the United Kingdom and United States and, most recently, in Canada, Australia, and Europe.

In this study, we investigated the behavior change exhibited by users of Sweatcoin and analyzed the change in physical activity following engagement with the app. In addition, we used a survey from a subsample of users to identify which populations were most likely to show the biggest activity change.

We hypothesized that users who will be motivated to increase their physical activity following engagement with the app can be predicted through a range of demographic and other self-reported lifestyle variables.

## Methods

### The Sweatcoin App

**Figure 1:**
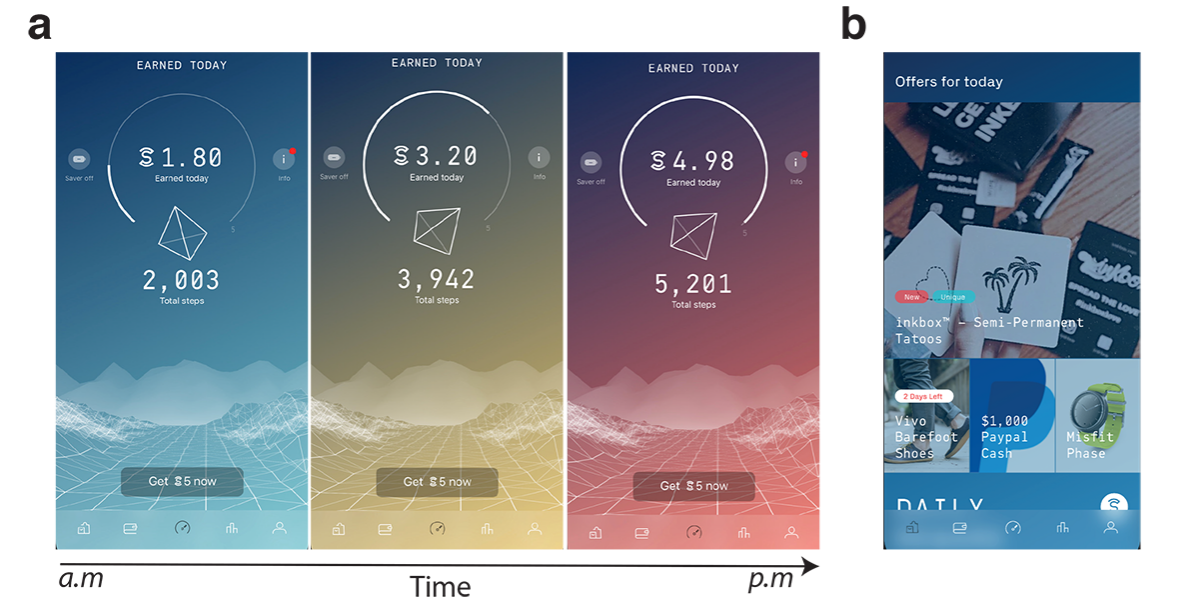
(a) Screenshots from the Sweatcoin app (as of December 2018). As a user’s step count (recorded by their smartphone) accumulates over the day, it is verified and converted into Sweatcoins. (b) The Sweatcoins are stored in the user’s wallet and can be subsequently used to purchase products, services, or subscriptions that are available on the marketplace.

Sweatcoin is a free app available on iOS and Android platforms. The concept of Sweatcoin is to convert a user’s step count, as recorded by the sensors on a smartphone, into virtual currency [11]. To ensure the value of the reward is maintained, the app incorporates a second layer of step verification. This operates by ensuring the distance moved (calculated from global positioning system [GPS] measurements) is representative of the number of steps recorded over a time period. Consequently, the app will convert validated outdoor steps (where a GPS signal is available) into Sweatcoins (Figure 1). Currently, 1000 steps will earn 0.95 Sweatcoins.

Users accumulate Sweatcoins that can subsequently be spent on the marketplace, accessible through the app (Figure 1). The marketplace usually has 3 to 4 daily offers and a similar number of *marathon* offers available. The daily offers are low cost and have short-term availability, with a new offer added each day (with the oldest product removed so that each product remains on sale for around 4 days). The offers can be for free products, services, or subscriptions. Sweatcoin currently has over 300 commercial partners who offer products and services on the marketplace. Marathon offers are a fixed set of high-value products (smartphones and televisions) that are priced such that it would take a user 12 to 18 months to accumulate enough Sweatcoins to make a purchase.

### Activity Dataset

A dataset containing daily step count for each user was used for the analysis of this study. The dataset contained no identifiable information, with the exception of a *Sweatcoin user identification number* such that only those internal to the company could identify which users were included in the analyses. The dataset was acquired through the Sweatcoin app from Apple iPhone users who had agreed to share their step count data, captured by Apple HealthKit, with the app. To ensure a consistent sample of user data, additional inclusion criteria were applied to the dataset. In particular, we only collated data from users who had daily step count data for a minimum of 6 months after originally registering with Sweatcoin, and an additional 3 months data before registration. It was important that we only analyzed data from users who were engaging with the app to get the best assessment of the app’s impact on physical activity change. Therefore, users included in the dataset also had to have opened the app within the last week.

**Figure 2:**
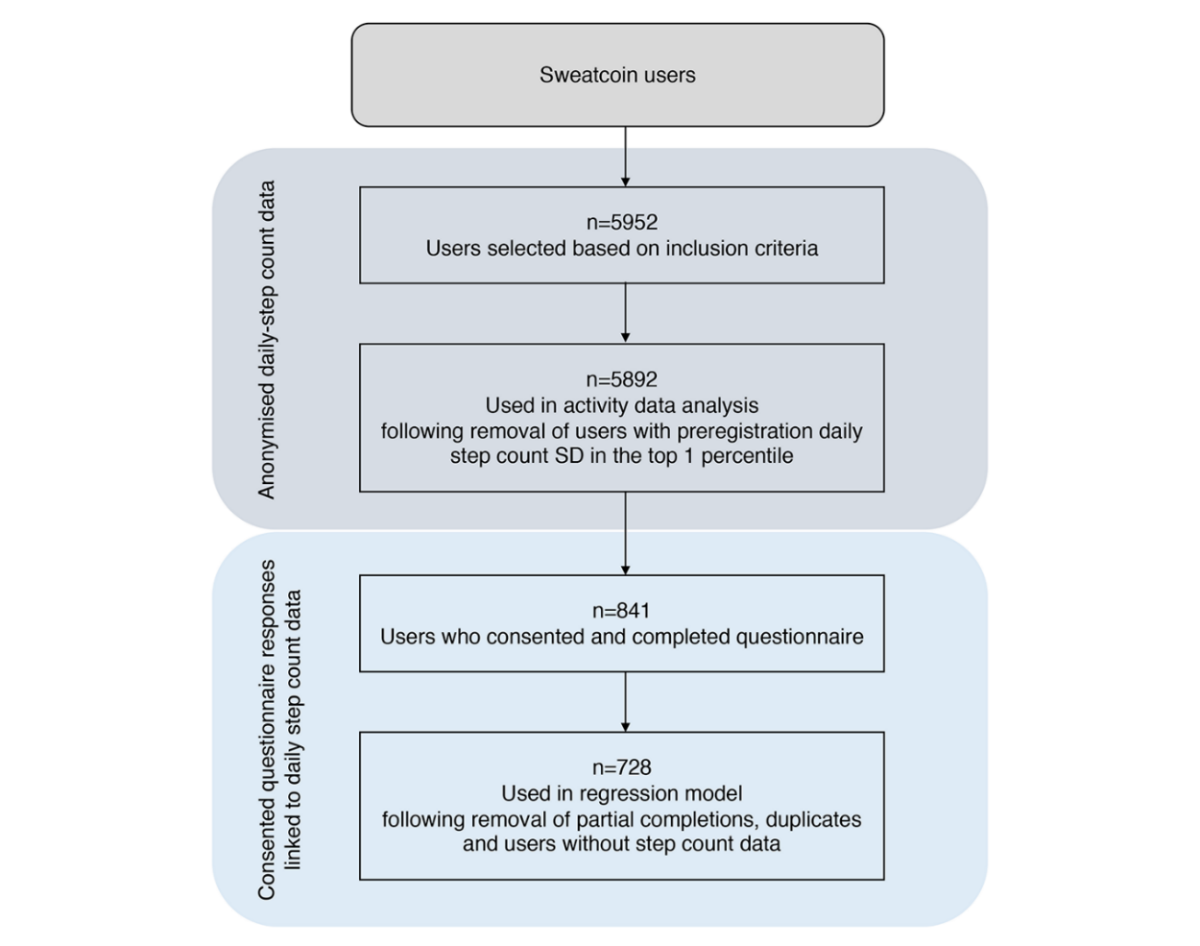
Flowchart of analysis stages with corresponding sample sizes.

On the basis of these inclusion criteria, 5952 Sweatcoin users were included in the daily step count dataset (Figure 2). The data were split approximately equally between UK and US residents. The raw data consisted of a unique Sweatcoin user identification number, date of registration, number of steps recorded (for 1 day), and the date of the recording.

### Activity Data Processing

Processing and analysis of the activity data were completed using R (version 3.4.1), a programming language specifically designed for statistical data analysis [12]. For each user, the daily step count data were split into the days occurring before Sweatcoin registration and the days occurring after registration. Additional fields of weekday versus weekend and season were added to each entry based on the date of the recorded steps. Similarly, the data were subgrouped into monthly (30-day) periods relative to registration, with negative months (−1 through −3) reflecting periods before registration and positive months (1 through 6) reflecting the period after registration. It should be noted that there was no *month 0*; the date of registration was defined as the first day of month 1. Any entries that were more than 90 days before registration or 180 days after registration were discarded.

For each 30-day period within each user’s data, we calculated the mean, median, maximum, minimum, upper quartile, lower quartile, and SD of the daily step counts. However, for this study, we focused only on the mean values recorded. In addition to the mean daily step count recorded over the whole 30-day period, we further measured the mean value across all weekend days (Saturdays and Sundays only) occurring within the 30-day period along with the mean of the weekdays only (Monday through Friday) in that period.

### Physical Activity Change Analyses

The aim of the initial analysis was to quantify the change in physical activity (in the context of daily step count) after registering with the Sweatcoin app, relative to the 3-month period before registration. We did this by taking the mean across months −1 to −3 and subtracting this value from the mean daily step count of all 9 months. This resulted in a relative daily step count that was centered around zero for the period before registration and quantified any increase or decrease in the 6 months after registration. These relative step counts for the period after registration were subsequently converted to a percentage of the mean daily step count measured across months −1 to −3 to normalize against the large variation in activity levels across the sample.

We excluded users who had highly variable step counts in the 3-months preregistration period as the variability would impact the calculations of activity change in the postregistration period. This was achieved by ranking users according to the SD of their mean steps per day across months −1 to −3 and excluding those in the top percentile (equating to a SD >4020 steps/day). The final sample size for analysis was 5892 after removing these users (Figure 2).

Users were finally classified into groups according to their level of *physical activity behavior change* following registration of the app, measured as the mean percentage change over 6 months. Overall, 3 groups were defined: no or negative change, where the average percentage change was zero or less; moderate change, where the average percentage increase was greater than zero, but less than or equal to the overall mean observed across the sample (18.7%, see Results); and high change, where the average percentage increase was greater than the overall mean observed across the sample.

### Demographics Questionnaire

On the basis of the grouping of users according to their change in activity following app registration, we wanted to determine if there were any demographic differences between these groups. To achieve this, we defined a set of demographic variables of interest to be tested (Table 1).

**Table 1:** Demographic and other variables included in the questionnaire, along with options or categories.

Variable	Options or categories
Gender	Male, Female
Age	<18 years [excluded], 18-24 years, and then 10-year increments up to 85 years or older
Height	1 m or less, increments of 10 cm up to 2 m. Equivalent feet and inches measurements also shown
Weight	40 kg or less, increments of 10 kg up to 120 kg, or >120 kg. Equivalent stones and pounds measurements also shown
Education	High school (or equivalent), Grammar school, College degree, Bachelor’s degree, Master’s degree, Professional degree, Doctoral degree, or Other
Employment	Student, Retired, Unemployed, Homemaker, Self-Employed, Private sector, or Public sector
Income	<£10,000, then £10,000 increments up to £100,000, £100,000-£149,999, £150,000-£200,000, over £200,000, or would rather not say. (Equivalent US dollar amounts also shown.)
Marital status	Living with another, Married/civil partnership, Separated, Divorced, Widowed, Would rather not say, or Other
Dog owners	Yes, No
Have children	Yes, No
Regularly use a wearable fitness tracker	Yes, No
Home location	Urban, Suburban, or Rural
Commute type	Car, Bus, Train, Tram/Tube, Walk, Cycle, or Other/None
Commute distance	No commute/no fixed place of work, <5 km, 5-10 km, 11-15 km, 16-20 km, 21-30 km, or >30 km
Motivations to exercise	Respondents chose one option from: Increase my overall health, Lose weight, Gain strength, Improve my skills, Have fun, Spend time with friends, To look good, or Other
Self-reported physical activity	On the basis of the General Practice Physical Activity Questionnaire [13], respondents state level of activity at work (multiple choice, from “most of time sitting” through to “vigorous physical activity”) and number of hours (between 0 and 3 or more) spent on a range of different activities in the last week
Rigidity score	On the basis of the compulsive exercise test [14], respondents state how often each of 4 statements is true, ranging from never to always on a 6-point Likert scale. The statements cover organization and structure of activities
Walking pace	Slow (<3 mph), Steady, Brisk, or Fast (>4 mph)
Other health/fitness apps used	Respondents chose from Never, Sometimes, or Regularly from the following apps: 7-Minute Workout, 8fit Planner, Calm Meditation, Calorie Counter, Fitbit, Headspace, MyFitnessPal, Nike, Strava, and Weight Watchers

### Recruitment

To recruit for the questionnaire survey, all users included in the original sample were shown an advert on the Sweatcoin marketplace inviting them to participate. Users who registered to participate were provided a link to the questionnaire (hosted on the Google Forms platform). Participant information and ethical approval information were provided on the first screen. Consent was registered by participants explicitly ticking a box to register their consent to take part and then continuing to the questionnaire. Those who completed the questionnaire were sent a £10 (United Kingdom) or US $10 retail voucher. The questionnaire typically took participants around 10 min to complete.

Over a period of 4 weeks, a total of 841 users completed the questionnaire (Figure 2). Of these, 728 users remained after removing partial completions, duplicates, and users who were not in the original activity dataset (Figure 2). The corresponding daily step count data were then linked to the resulting sample of questionnaire responses.

### Analysis

The questionnaire data were coded and restructured as required to make each variable suitable for regression analysis. Dummy variables were created for multiple-choice responses. Self-reported activity and rigidity scores were calculated as per guidelines. However, it should be noted that rather than limiting the General Practice Physical Activity Questionnaire (GPPAQ) score to a maximum of 3, we used the total score from all activities. In addition to the questionnaire variables, the season of registration was added as a further set of dummy variables to ensure seasonality would be accounted for in the analysis.

The physical activity change class (Table 2) was used as the dependent variable in a multinomial regression, with the questionnaire and other variables described above used as the independent variables. Using this model, we were able to investigate the predictor variables that differentiated the moderate and high behavioral change groups from the no or negative change group. A total of 728 participants were included in the regression analysis (Figure 2). All variables were entered simultaneously into the regression (see Multimedia Appendix 1 for full regression results including nonsignificant variables).

**Table 2:** Classification of physical activity behavior change based on percentage change in daily step count relative to the 3 months preregistration period. The number of samples in each class is shown for both the daily step count data and the combined questionnaire responses with daily step count.

Class	Label	Range, %	Number of users in class, n (%)	
			Activity dataset (N=5892)	Questionnaire responses (N=728)
0	No or negative change in activity	<1	2172 (36.86)	258 (35.4)
1	Moderate positive change in activity	1-18.7	1542 (26.17)	196 (26.9)
2	High positive change in activity	>18.7	2178 (36.96)	274 (37.6)

### Ethical Approval

Ethical approval was received from the University of Warwick Biomedical and Scientific Research Ethics Committee (Approval Number: REGO-2017-2086). In addition, 2 members of the academic research team received honorary contracts with Sweatcoin to oversee the analysis of the anonymized activity data. Recruitment for the demographic questionnaire was further handled by employees of Sweatcoin to ensure participants’ activity data were not made identifiable outside of the company until they had provided consent to merge their activity data with the questionnaire responses.

## Results

### Change in Daily Step Count Following App Registration

**Figure 3:**
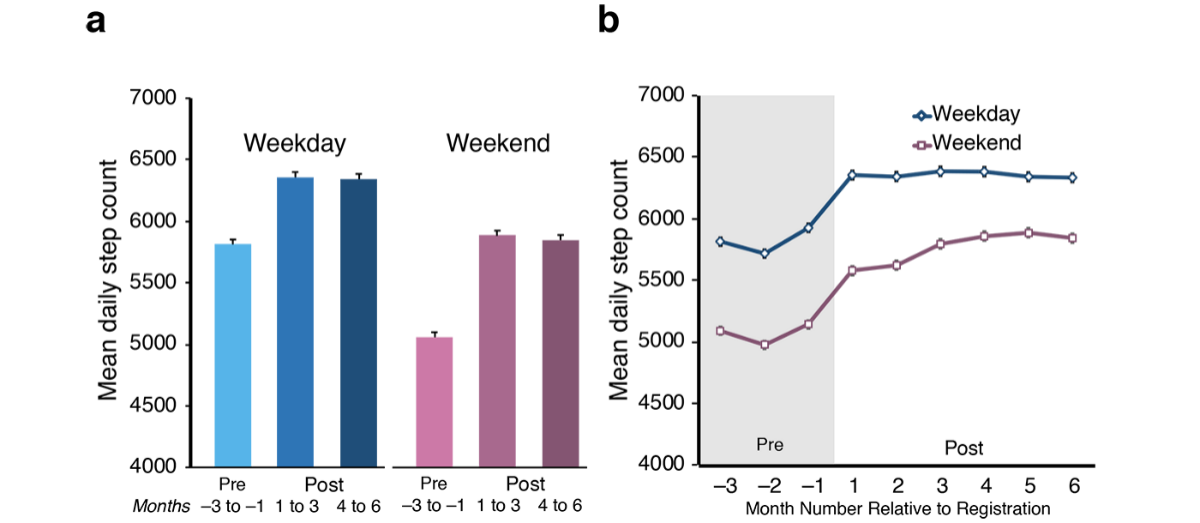
Mean daily step count across the sample (N=5892) was analyzed for the 3 months before app registration (Pre) and 6 months after registration (Post). (a) For analysis, these were grouped into 3-month periods; the results highlight the consistent increase in mean daily step count after registration. (b) The same measure is broken down over 30-day periods. Both plots show the data separated into weekday and weekend activity. Although weekend activity is lower, the overall pattern of increase after registration is similar. Error bars represent SE of the mean.

Initially, mean daily step count was examined across three 3-month periods. In particular, we contrasted the mean daily step count for the 3 months before registration with the app and for two 3-month periods following app registration (months 1-3 and 4-6; see Figure 3). This was further split into both weekday and weekend activity. Overall activity levels were higher during the week than at weekends. Importantly, we found a significant rise in daily step count following app registration for both weekend and weekday activities, with weekend step counts showing a greater increase than those on weekdays (interaction effect, *F*_2,11782_=101.49; *P*<.001). Post hoc analysis confirmed that the increase remained consistent over the 6-month period, with no significant difference between months 1-3 and months 4-6 following app registration for either weekdays (*P*>.99) or weekends (*P*=.30).

**Figure 4:**
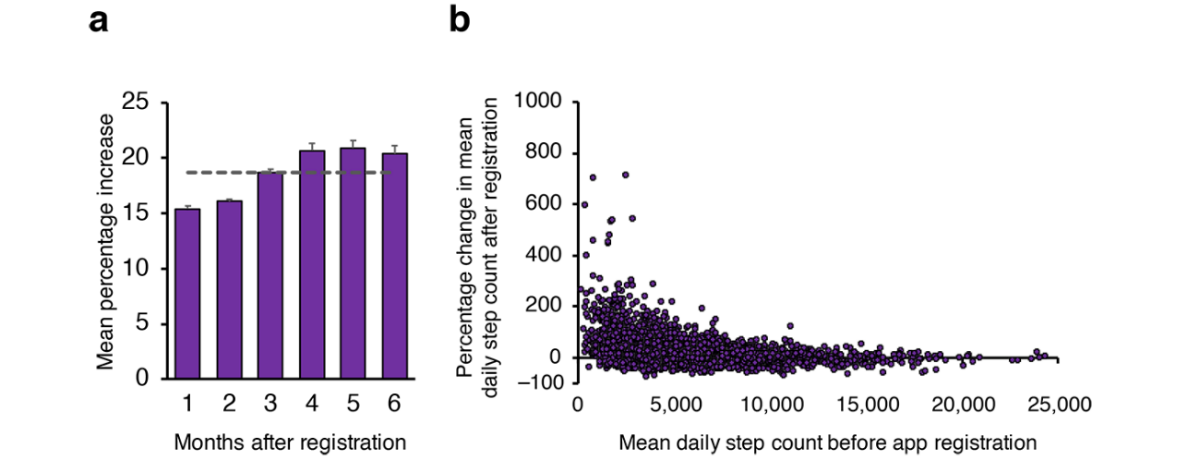
(a) Percentage change in daily step count for the period after registration, relative to the 3 months before registration (N=5892). The horizontal dashed line (gray) represents the overall average percentage increase of 18.7%. Error bars represent SE of the mean. (b) Individual data points (N=5892) of mean daily step count before app registration against the subsequent percentage change after registration.

For the remaining analyses, we no longer contrasted weekend and weekday activity and, hence, averaged the daily step count across continuous 30-day periods. The relative percentage activity change following app registration was calculated as described in the Methods section, revealing an overall mean increase of 18.7% (47.9%) in daily step count across the sample (Figure 4). Across the 6 months, this increase ranged from 15.4% (month 1) up to 20.9% in month 5. Plotting the percentage change against the mean daily step count across the 3 months before registration (Figure 4) reveals that those who were less physically active (in terms of step count) before using the app had a higher range of increased activity after registration. Individuals who were already physically active tended to show minimal further increase following registration. Reduced levels of physical activity after registration (negative percentage change) did not appear to vary according to previous levels of activity.

### Classification Thresholds

From the overall mean activity change of 18.7% across 6 months, we defined user activity behavior change classifications as shown in Table 2. Users were then split into these classes based on their overall activity change for the 6 months after registration.

### Seasonality Effects

**Figure 5:**
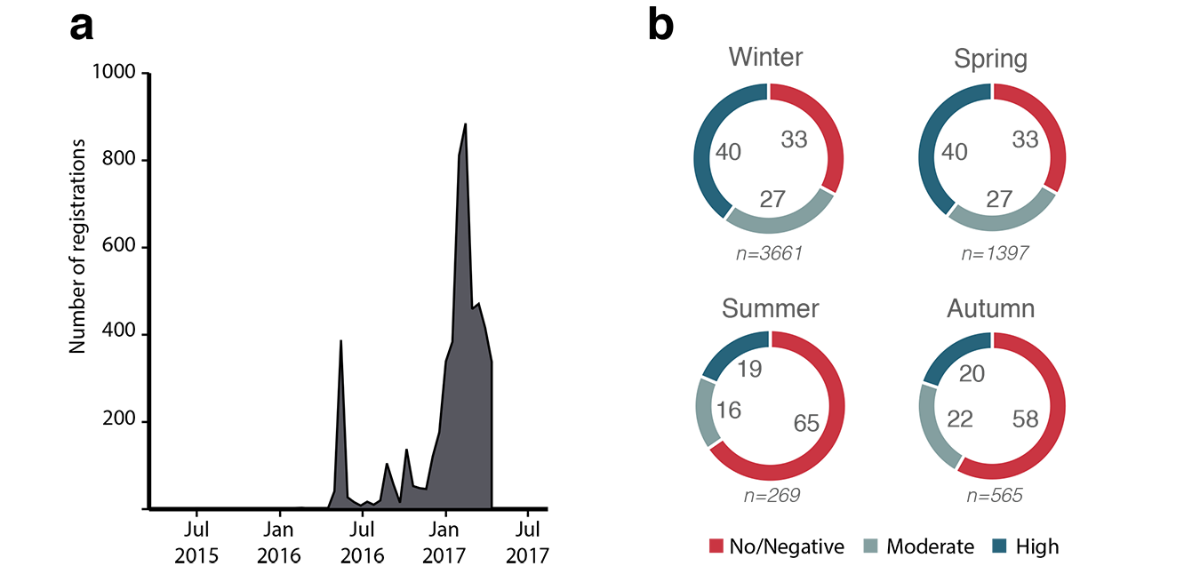
(a) Histogram showing distribution of user registrations in the sample data (N=5892). The main app launch can be identified in May 2016, whereas there is a further rapid acceleration around December 2016, which skews the data toward the winter and spring seasons. (b) User classifications of activity behavior change broken down across seasons; numeric values inside the circles represent the percentage of users in the associated class; sample sizes for each season are shown in italics under each chart. For winter and spring, there is a higher proportion of high activity change users than no or negative change users. Summer and autumn show an over representation of no or negative change users.

Figure 5 shows the distribution of user registrations for the dataset analyzed and highlights the accelerating rate of registration over a short period of time. This provides a relatively skewed dataset in terms of most people registering over the winter or spring period. Seasonality effects were investigated to examine the distribution of registrations among classes. This was to evaluate the risk that activity change classifications were based primarily on season rather than engagement with the app. For the winter and spring data (when the majority of users registered) we observed that, despite a relatively even split of classifications, there is a greater proportion of high activity change users than no or negative change users (Figure 5). In contrast, summer and autumn registrations are more over-representative of no or negative change users. However, the overall sample from these 2 latter seasons was much smaller. The season of registration of each user was also added as a dummy variable to the later regression model to ensure any seasonality was accounted for in the analysis.

### Demographic Survey Profile

In Table 3, the descriptive statistics of the main demographic variables are listed. In addition, the sample was split evenly geographically, with 49.6% of respondents from the United Kingdom and 50.4% from the United States. Measures of exercise rigidity [14] were low on average, with means of 2.40 (like days to be organized and structured), 2.25 (pattern of exercise is repetitive), and 2.12 (set routine for exercise). In addition, respondents were asked about which other fitness-related apps they used, either occasionally or on a regular basis (Table 4).

**Table 3:** Descriptive statistics of key variables in the questionnaire (N=728).

Variable		Frequency, %
**Gender**		
	Male	64.7
	Female^a^	35.3
**Age (years)**		
	18-24	39.7
	25-34	38.9
	35-44	17.3
	Over 45^a^	4.1
**Weight status (based on body mass index estimate)**		
	Underweight	3.2
	Healthy weight^a^	49.2
	Over weight	21.6
	Obese	26
**Has a degree**		
	Yes	67.7
	No^a^	32.3
**Employment status**		
	Not in employment	6
	Student	29
	Employed (private sector)^a^	38.3
	Employed (public sector)	19.4
	Self-employed	7.3
**Annual household income**		
	Less than £20k	17.4
	£20k-£39k	22.4
	£40k-£59k^a^	33.7
	£60k-£79k	12.8
	£80k or more	13.7
**Marital status**		
	Married/civil partnership	27.2
	Cohabiting	15.7
	Divorced/separated	4.3
	Single^a^	52.8
**Have a dog**		
	Yes	31
	No^a^	69
**Have children**		
	Yes	31.9
	No^a^	68.1
**Use a wearable device**		
	Yes	46.4
	No^a^	53.6
**Home location**		
	Urban^a^	38.2
	Suburban	44.4
	Rural	17.4
**Commute/transport type**		
	Walk	19.6
	Cycle	4.3
	Bus	9.5
	Car	46.8
	Train	6.6
	Tube/tram	7.4
	Other	5.8
**Commute distance**		
	None/no fixed location	10.7
	<5 km	28.3
	5-10 km	22
	11-15 km	11.4
	16-20 km	8.9
	21-30 km	7.8
	>30 km	10.9
**Motivation to exercise**		
	Increase overall health^a^	37.4
	Lose weight	29.8
	Gain strength	12.1
	Look good	10.9
	Improve skills	3.2
	Have fun	4.3
	Spend time with friends	2.3
**Activity level (self-reported GPPAQ^b^** **score [13])**		
	Inactive	13.3
	Moderately inactive	13.6
	Moderately active	19.8
	Active	53.3
**Walking pace**		
	Slow (<3 mph)	7.1
	Steady	44.5
	Brisk	38.6
	Fast (>4 mph)	9.8

^a^Response options were used as the reference response with which the other responses for that variable were compared in the regression model.

^b^GPPAQ: General Practice Physical Activity Questionnaire.

**Table 4:** Frequency of usage of other fitness- and well-being–related apps (N=728).

App	Never use, %	Sometimes use, %	Regularly use, %
7-Minute Workout	76.1	19.2	4.7
8fit Planner	88.7	8.1	3.2
Calm Meditation	76.2	17.7	6.0
Calorie Counter	81.6	12.9	5.5
Fitbit	72.1	10.2	17.7
Headspace	79.8	15.0	5.2
MyFitnessPal	60.0	25.3	14.7
Nike	74.0	18.1	7.8
Strava	77.7	11.1	11.1
Weight Watchers	88.6	7.3	4.1

### Predictors of Activity Behavior Change

On the basis of the classification of users according to their change in activity following app registration, we investigated the main predictors of this behavior change based on the demographic questionnaire variables. A multinomial logistic regression was run to compare users in the moderate and high activity change classes with respect to those in the no or negative activity change class. Independent variables included the demographic and other variables captured by the questionnaire (see Tables 3 and 4).

The results (N=728, χ^2^_146_=197.6 *P*=.003; Nagelkerke pseudo R^2^=26.8%) report the predictors of both moderate change and high change with respect to no or negative change. Significant results are reported in Table 5 (moderate) and Table 6 (high) with associated ORs and significance levels; full results are provided in Multimedia Appendix 1. The seasonal skew identified above was present for both moderate and high change groups, with those registering in autumn (OR 4.17; *P*=.04), winter (OR 11.54; *P*<.001), and spring (OR 10.88; *P*=.001) seasons being more likely to show a moderate increase in activity after downloading the app, relative to those registering in summer. Similarly, those exhibiting high levels of activity increase were more likely to have registered in winter (OR 4.67; *P*=.001) or spring (OR 5.05; *P*=.001). After accounting for seasonality, a further significant predictor variable was found, with regular users of MyFitnessPal being significantly less likely to show moderate activity change (relative to no or negative change). For the high change group, users were more likely to be classed as *overweight* (relative to *normal weight*, calculated using body mass index) compared with those in the no or negative change group (OR 1.83; *P*=.02). Similarly, those with lower self-reported activity levels were more likely to be in the high change group (OR 0.88; *P*=.048). We also noted a slight positive relationship between rigidity score and high behavior change (OR 1.07; *P*=.02).

**Table 5:** Multinomial logistic regression results for predictors of moderate activity behavior change classification versus no or negative activity change (N=728). Results show the odds ratios of the significant predictor variables (*P*<.05). Odd ratio values less than 1 represent negative relationships.

Variable	Model coefficients (B)	SE	Wald	Odds ratio	*P* value
Registered in winter	2.45	.67	13.36	11.54	<.001
Registered in spring	2.39	.69	11.92	10.88	.001
Registered in autumn	1.43	.71	4.05	4.17	.04
Regular user of MyFitnessPal	−0.89	.37	5.77	0.41	.02

**Table 6:** Multinomial logistic regression results for predictors of high activity behavior change classification versus no or negative activity change (N=728). Results show the odds ratios of the significant predictor variables (*P*<.05). Odds ratio values less than 1 represent negative relationships.

Variable	Model coefficients (B)	SE	Wald	Odds ratio	*P* value
Registered in winter	1.54	.46	11.13	4.67	.001
Registered in spring	1.62	.49	11.08	5.05	.001
Overweight	0.61	.27	5.18	1.83	.02
Rigidity score	0.07	.03	5.72	1.07	.02
GPPAQ^a^ score	−0.13	.07	3.90	0.88	.048

^a^GPPAQ: General Practice Physical Activity Questionnaire.

## Discussion

In this study, we have investigated physical activity behavior change in users engaging with an app that converts step count into virtual currency (Sweatcoins). Using an initial sample of 5892 users who had been registered with the app for 6 months or longer, we used anonymized daily step count data to analyze activity levels for the 6-month period following registration and compared it with that recorded for the 3-month period before registration. Importantly, our results found a significant and consistent increase in step count following app registration, which averaged 18.7% across the period and across all users. In an earlier pilot analysis, we previously reported a figure of 19.5% because of a slight variation in inclusion criteria [11].

The respondents to the subsequent questionnaire were mainly from the young adult population, which is likely to be representative of Sweatcoin’s overall user demographic. Therefore, our results do not necessarily generalize across all age groups. However, the captured demographic is an important generation to target, with many physical activity programs focusing specifically on children or older adults but few targeting young adults and capitalizing on their engagement with smartphone technologies [15]. Therefore, there is potential for this app to be used as an intervention aimed at young and middle-aged adults who are at an important transition period for weight gain and development of obesity [16].

The classification of users into levels of activity behavior change and subsequent demographic survey data allowed us to examine in detail which users are most likely to increase activity following registration with the app. Despite the wide range of variables captured, the equivalent variance accounted for by the logistic regression model was relatively low at 26.8%, highlighting that there are likely additional lifestyle events not captured that could contribute to increased activity behavior. In particular, we did not capture any medical conditions the respondents may have had at the time that may impact the levels of physical activity. Weather-based effects [17,18] and holiday periods [19] can also have a strong influence on an individual’s activity levels. Although it was not practical to factor in daily weather patterns, seasonality was included in our regression model, with winter and spring showing as strong predictors of activity change. This is most likely to relate to an effect of *New Year’s Resolutions* creating an uplift in activity. On the basis of the change in activity observed that aligned with the registration with the app (Figure 3), it is likely that users were using the rewards as a motivational tool during this period.

Taking seasonality into account, we found additional variables that distinguished moderate and high activity change groups from those showing no or negative change. Regular users of MyFitnessPal were significantly less likely to be in the moderate change group compared with the no or negative change. MyFitnessPal [20] is primarily a dietary monitoring app where users can accurately measure food intake, and it was the second most regularly used app by the Sweatcoin users surveyed (Table 4). It is possible that users of this app are more focused on changing their food intake behavior than increasing physical activity levels, although we did not observe a similar relationship in the high change group.

For the high change group, we found that a number of variables were significant predictors of this group relative to no or negative change. We found a small positive relationship to a user’s rigidity score [14], suggesting that those with a more structured lifestyle and exercise routine were more likely to increase activity behavior. Importantly, users classified as *overweight* and self-reporting lower levels of physical activity were also more likely to be in the high change group. Therefore, the motivation to increase physical activity based on the rewards offered by the app was greatest in a group of users who were inactive, highlighting that the app was motivating the target demographic of sedentary individuals to become more active. The relationship between the mean daily step count before app registration and percentage change after registration also highlights this, with those with already high step counts showing little increase after registration (Figure 4). We cannot totally rule out that our measure is showing some *regression to the mean* effects, where the extreme low and high values measured in the preregistration phase are naturally more likely to be closer to the average in a follow-up phase. However, although we observed higher positive change in those with low daily step count before registration, we did not see a correspondingly large negative shift after registration in those who initially had a very high step count. In addition, the predictor variables that were significant in the high change group indicate self-reported sedentarism (ie, lower GPPAQ score and overweight), suggesting those users were not just captured in an unusually inactive period before app download. Therefore, we suggest that those who are already active will accumulate rewards at a reasonable rate anyway, and hence, the extrinsic motivation is limited relative to their intrinsic motivation levels to exercise, similar to that observed with fitness tracker usage [21]. Individuals who are defined as obese (rather than overweight) are more likely to require more formal interventions to change physical activity behavior. As such, individuals who are overweight, but not obese, appear to be those who can most benefit from incentive-based physical activity programs because of low intrinsic motivation, but with minimal physical barriers to increasing activity levels.

The overall consistent increase in physical activity over the 6-month period following registration highlights a differentiation from other incentive-based and goal-oriented programs reported, which typically show only short-term behavior change effects [10]. A common cause of people failing to maintain physical activity over the long term is the fading novelty effect of activity trackers and apps. Most fitness trackers allow users to set goals and compete with other users, providing *badges* and other virtual rewards once a goal has been achieved. These incentives to change behavior are successful in the short term, but over one-third of US citizens stop using fitness trackers within 6 months of purchase [22]. In a similar example, Pokemon GO, an app-based game that requires users to search out rewards in specific geographic locations, resulted in a large increase in activity levels [23]. However, users reverted to previous activity levels after just 6 weeks. The Sweatcoin concept in this study allows users to be continuously and immediately rewarded for their activity. Although a reduction in novelty over time is still a risk, the effect is minimized by daily rotations of products available on the marketplace. In addition, a wide range of product types and prices allow users to make regular purchases of low-cost items or save toward larger purchases. The results we have reported suggest this concept leads to a maintained increase in physical activity over a 6-month period.

Monetary and other tangible incentives have been effective in increasing physical activity behavior in sedentary populations [9,10,24]. However, often, incentive programs are only funded for a short period either because of being trial-only programs or because of limited funding available to provide the rewards. Incentives provide an extrinsic motivation to exercise, and therefore, unless an individual develops intrinsically motivated attitudes to exercise [7] (ie, self-motivated) within the period that rewards are offered, participants are at risk of returning to their previous behavior once the Sweatcoin app has developed a sustainable business model, where users are continuously generating their own virtual currency that companies are willing to accept in exchange for their goods and services (because of the exposure to a large customer base). As the Sweatcoin platform continues to grow, the associated datasets will increase timewise, and future analyses could investigate incentive-based engagement over much longer periods—something that has not been possible with other programs.

There are some limitations to this study. In particular, we were unable to establish a control group as a direct comparator to the intervention group we analyzed. This would have required the recruitment of a similar sample (ie, N>5000) of non-Sweatcoin app users who would share their daily step count data over matched 9-month periods. However, the dataset we used included both pre- and postregistration data, such that the preregistration data provided an objective baseline measure of activity. Furthermore, during the period that the data were generated, users were unaware that it would be used for this kind of analysis, and hence, there was no risk of experimental bias in the data.

There was also risk of bias being introduced because of sampling of users. We only used data from iPhone users because of the limitation of the app only being able to access daily step count data via Apple HealthKit on these devices. This could have potentially skewed the demographic profile of the sample recruited, although it has recently been reported that there are few demographic and personality differences between iOS and Android users [25]. We further only recruited users that had recently opened the app. This was to ensure we were only analyzing engaged users and, hence, measuring the effect of incentives as much as possible (ie, avoiding users who may have downloaded the app, opened it once and never opened again). It is possible that there was some overlap between a user that is engaged with the app and their motivation to increase their exercise. However, the distribution of users across the different groupings of activity change was fairly equal. Similarly, bias could have been introduced in the users selecting to complete the survey, again recruiting those more motivated to change. However, the distribution of users in the activity change groups in the survey sample was almost identical to those in the original activity dataset (Table 2), suggesting that recruitment did not bias the sample, at least in terms of level of activity change.

In conclusion, the Sweatcoin concept allows users to continuously be incentivized to be physically active through generation of virtual currency from steps. Through analysis of a sample of Sweatcoin users, we have observed a sustained increase in physical activity (measured by daily step count) over a 6-month period, with users identified as overweight and less active most likely to show the highest increases in activity after registering with the app.

## Appendix

Multimedia Appendix 1 Full regression results.

## Abbreviations

BMI: body mass index
GPPAQ: General Practice Physical Activity Questionnaire
GPS: global positioning system
OR: odds ratio
