# Supplementary material for: Physical Activity Behavior Change Driven by Engagement With an Incentive-Based App: Evaluating the Impact of Sweatcoin
Source: JMIR Mhealth Uhealth. 2019 Jul 8;7(7):e12445. doi: 10.2196/12445 (PMC6643767; doi:10.2196/12445)
Supplement: Multimedia Appendix 1 [file mhealth_v7i7e12445_app1.pdf]

Full multinomial regression results for Moderate vs No/Negative change

Independent Variable	Comparator Variable	B	Std. Error	Wald	df	Significance	Odds Ratio
Intercept	N/A	-1.391	1.155	1.45	1	0.228	
GPPAQ Score	N/A	-0.03	0.07	0.184	1	0.668	0.971
Rigidity Score	N/A	0.046	0.031	2.171	1	0.141	1.047
UK Resident	US Resident	0.032	0.26	0.015	1	0.903	1.032
Not a dog owner	Dog owner	-0.186	0.254	0.539	1	0.463	0.83
No children	Has children	-0.296	0.262	1.275	1	0.259	0.744
Walking Pace: Slow	Walking Pace: Fast	-1.122	0.585	3.67	1	0.055	0.326
Walking Pace: Steady	Walking Pace: Fast	-0.682	0.379	3.236	1	0.072	0.506
Walking Pace: Brisk	Walking Pace: Fast	-0.384	0.375	1.048	1	0.306	0.681
Gender: Female	Gender: Male	0.405	0.254	2.54	1	0.111	1.499
Rural Home Location	Urban Home Location	-0.358	0.343	1.087	1	0.297	0.699
Suburban Home Location	Urban Home Location	-0.314	0.252	1.553	1	0.213	0.73
Has a degree	Does not have a degree	-0.24	0.261	0.846	1	0.358	0.787
Commute Distance: Work from Home	Commute Distance: Over 30km	-0.279	0.578	0.234	1	0.629	0.756
Commute Distance: <5km	Commute Distance: Over 30km	0.313	0.443	0.501	1	0.479	1.368
Commute Distance: 5-10km	Commute Distance: Over 30km	0.055	0.393	0.019	1	0.889	1.056
Commute Distance: 11-15km	Commute Distance: Over 30km	-0.306	0.469	0.428	1	0.513	0.736
Commute Distance: 16-20km	Commute Distance: Over 30km	0.509	0.475	1.15	1	0.283	1.664
Commute Distance: 21-30km	Commute Distance: Over 30km	-0.907	0.522	3.021	1	0.082	0.404
Underweight	Normal weight	0.42	0.656	0.41	1	0.522	1.522
Overweight	Normal weight	0.228	0.294	0.603	1	0.437	1.256
Obese	Normal weight	0.159	0.288	0.303	1	0.582	1.172
Age 18-24	Age 45 and Over	-0.268	0.71	0.142	1	0.706	0.765
Age 35-44	Age 45 and Over	0.45	0.666	0.456	1	0.499	1.568
Age 25-34	Age 45 and Over	0.141	0.656	0.046	1	0.829	1.152
Salary £0-£20k	Salary £40-£60k	0.492	0.378	1.688	1	0.194	1.635
Salary £20-£40k	Salary £40-£60k	0.118	0.32	0.136	1	0.712	1.125
Salary £60-£80k	Salary £40-£60k	0.31	0.37	0.7	1	0.403	1.363
Salary Above £80k	Salary £40-£60k	0.137	0.374	0.135	1	0.713	1.147
Not Stated	Salary £40-£60k	0.101	0.437	0.054	1	0.817	1.107
Employment Status: Student	Employment Status: Private Sector	-0.462	0.344	1.806	1	0.179	0.63
Employment Status: Not In Employment	Employment Status: Private Sector	-0.776	0.71	1.194	1	0.274	0.46
Employment Status: Self-Employed	Employment Status: Private Sector	-0.362	0.462	0.615	1	0.433	0.696
Employment Status: Public Sector	Employment Status: Private Sector	-0.151	0.306	0.244	1	0.622	0.86
Commute: Walk	Commute: Car	0.124	0.369	0.113	1	0.737	1.132
Commute: Cycle	Commute: Car	-0.654	0.63	1.076	1	0.3	0.52
Commute: Train	Commute: Car	0.293	0.455	0.414	1	0.52	1.34
Commute: Bus	Commute: Car	-0.042	0.401	0.011	1	0.916	0.958
Commute: Tube	Commute: Car	-0.565	0.481	1.378	1	0.24	0.568
Commute: Other/None	Commute: Car	-0.651	0.714	0.833	1	0.361	0.521
Marital Status: Divorced/Separated	Marital Status: Single	-0.349	0.684	0.261	1	0.61	0.705
Marital Status: Married	Marital Status: Single	-0.183	0.333	0.303	1	0.582	0.832
Marital Status: Cohabit	Marital Status: Single	0.331	0.327	1.026	1	0.311	1.392
Motivation: Lose Weight	Motivation: Increase Health	-0.138	0.279	0.246	1	0.62	0.871
Motivation: Gain Strength	Motivation: Increase Health	0.287	0.367	0.61	1	0.435	1.332
Motivation: Improve Skills	Motivation: Increase Health	-0.281	0.594	0.223	1	0.637	0.755
Motivation: Have Fun	Motivation: Increase Health	-0.886	0.651	1.852	1	0.174	0.412
Motivation: Time with Friends	Motivation: Increase Health	-0.162	0.74	0.048	1	0.827	0.851
Motivation: Look Good	Motivation: Increase Health	-0.554	0.383	2.085	1	0.149	0.575
**Registration Season: Spring**	**Registration Season: Summer**	**2.387**	**0.691**	**11.922**	**1**	**0.001**	**10.881**
**Registration Season: Autumn**	**Registration Season: Summer**	**1.428**	**0.709**	**4.054**	**1**	**0.044**	**4.171**
**Registration Season: Winter**	**Registration Season: Summer**	**2.446**	**0.669**	**13.357**	**1**	**0**	**11.542**
Do not use a wearable	Use a wearable	-0.134	0.248	0.293	1	0.588	0.875
Use 7min Workout Regularly	Never use this app	-0.415	0.686	0.366	1	0.545	0.66
Use 7min Workout Sometimes	Never use this app	-0.344	0.317	1.178	1	0.278	0.709
Use 8Fit Planner Regularly	Never use this app	1.332	0.991	1.807	1	0.179	3.788
Use 8Fit Planner Sometimes	Never use this app	0.301	0.537	0.315	1	0.575	1.352
Use Calm Meditation Regularly	Never use this app	-0.308	0.636	0.234	1	0.629	0.735
Use Calm Meditation Sometimes	Never use this app	-0.002	0.35	0	1	0.996	0.998
Use Calorie Counter Regularly	Never use this app	-0.592	0.638	0.863	1	0.353	0.553
Use Calorie Counter Sometimes	Never use this app	0.033	0.403	0.007	1	0.935	1.033
Use Fitbit Regularly	Never use this app	-0.099	0.346	0.081	1	0.775	0.906
Use Fitbit Sometimes	Never use this app	-0.307	0.412	0.557	1	0.456	0.735
Use Headspace Regularly	Never use this app	0.023	0.758	0.001	1	0.976	1.023
Use Headspace Sometimes	Never use this app	-0.418	0.37	1.274	1	0.259	0.658
**Use MyFitnessPal Regularly**	**Never use this app**	**-0.888**	**0.37**	**5.768**	**1**	**0.016**	**0.411**
Use MyFitnessPal Sometimes	Never use this app	0.007	0.27	0.001	1	0.978	1.007
Use Nike Regularly	Never use this app	0.811	0.451	3.244	1	0.072	2.251
Use Nike Sometimes	Never use this app	0.12	0.314	0.146	1	0.703	1.127
Use Strava Regularly	Never use this app	0.352	0.396	0.793	1	0.373	1.422
Use Strava Sometimes	Never use this app	0.08	0.389	0.043	1	0.836	1.084
Use WeightWatchers Regularly	Never use this app	-0.529	0.76	0.484	1	0.486	0.589
Use WeightWatchers Sometimes	Never use this app	1.005	0.561	3.212	1	0.073	2.731

Full multinomial regression results for High vs No/Negative change

Independent Variable	Comparator Variable	B	Std. Error	Wald	df	Significance	Odds Ratio
Intercept	N/A	-1.062	0.982	1.171	1	0.279	
**GPPAQ Score**	**N/A**	**-0.129**	**0.065**	**3.901**	**1**	**0.048**	**0.879**
**Rigidity Score**	**N/A**	**0.071**	**0.029**	**5.723**	**1**	**0.017**	**1.073**
UK Resident	US Resident	-0.28	0.239	1.37	1	0.242	0.756
Not a dog owner	Dog owner	-0.124	0.238	0.272	1	0.602	0.883
No children	Has children	-0.113	0.246	0.21	1	0.647	0.893
Walking Pace: Slow	Walking Pace: Fast	0.278	0.514	0.292	1	0.589	1.32
Walking Pace: Steady	Walking Pace: Fast	-0.146	0.37	0.156	1	0.693	0.864
Walking Pace: Brisk	Walking Pace: Fast	0.27	0.371	0.532	1	0.466	1.31
Gender: Female	Gender: Male	0.241	0.233	1.077	1	0.299	1.273
Rural Home Location	Urban Home Location	0.267	0.312	0.731	1	0.393	1.306
Suburban Home Location	Urban Home Location	0.439	0.234	3.522	1	0.061	1.551
Has a degree	Does not have a degree	0.065	0.241	0.072	1	0.788	1.067
Commute Distance: Work from Home	Commute Distance: Over 30km	-0.126	0.535	0.055	1	0.814	0.882
Commute Distance: <5km	Commute Distance: Over 30km	0.498	0.414	1.445	1	0.229	1.645
Commute Distance: 5-10km	Commute Distance: Over 30km	-0.026	0.383	0.005	1	0.946	0.974
Commute Distance: 11-15km	Commute Distance: Over 30km	0.441	0.425	1.077	1	0.299	1.555
Commute Distance: 16-20km	Commute Distance: Over 30km	0.447	0.461	0.944	1	0.331	1.564
Commute Distance: 21-30km	Commute Distance: Over 30km	0.096	0.448	0.045	1	0.831	1.1
Underweight	Normal weight	0.681	0.62	1.208	1	0.272	1.976
**Overweight**	**Normal weight**	**0.605**	**0.266**	**5.181**	**1**	**0.023**	**1.831**
Obese	Normal weight	-0.079	0.269	0.086	1	0.769	0.924
Age 18-24	Age 45 and Over	-0.784	0.582	1.814	1	0.178	0.457
Age 35-44	Age 45 and Over	-0.761	0.553	1.894	1	0.169	0.467
Age 25-34	Age 45 and Over	-0.571	0.528	1.17	1	0.279	0.565
Salary £0-£20k	Salary £40-£60k	0.099	0.336	0.087	1	0.768	1.104
Salary £20-£40k	Salary £40-£60k	-0.199	0.291	0.468	1	0.494	0.819
Salary £60-£80k	Salary £40-£60k	-0.162	0.351	0.213	1	0.645	0.851
Salary Above £80k	Salary £40-£60k	-0.424	0.343	1.524	1	0.217	0.655
Not Stated	Salary £40-£60k	-0.685	0.398	2.968	1	0.085	0.504
Employment Status: Student	Employment Status: Private Sector	-0.337	0.31	1.178	1	0.278	0.714
Employment Status: Not In Employment	Employment Status: Private Sector	0.092	0.59	0.024	1	0.876	1.096
Employment Status: Self-Employed	Employment Status: Private Sector	-0.101	0.435	0.054	1	0.816	0.904
Employment Status: Public Sector	Employment Status: Private Sector	-0.408	0.297	1.896	1	0.169	0.665
Commute: Walk	Commute: Car	0.347	0.347	1.005	1	0.316	1.415
Commute: Cycle	Commute: Car	0.13	0.52	0.063	1	0.802	1.139
Commute: Train	Commute: Car	0.372	0.445	0.7	1	0.403	1.451
Commute: Bus	Commute: Car	0.357	0.357	0.999	1	0.317	1.429
Commute: Tube	Commute: Car	0.4	0.406	0.971	1	0.324	1.492
Commute: Other/None	Commute: Car	-0.357	0.624	0.326	1	0.568	0.7
Marital Status: Divorced/Separated	Marital Status: Single	0.609	0.596	1.044	1	0.307	1.838
Marital Status: Married	Marital Status: Single	-0.142	0.311	0.208	1	0.649	0.868
Marital Status: Cohabit	Marital Status: Single	-0.167	0.317	0.277	1	0.599	0.846
Motivation: Lose Weight	Motivation: Increase Health	0.113	0.261	0.189	1	0.664	1.12
Motivation: Gain Strength	Motivation: Increase Health	0.52	0.343	2.295	1	0.13	1.682
Motivation: Improve Skills	Motivation: Increase Health	-0.002	0.59	0	1	0.997	0.998
Motivation: Have Fun	Motivation: Increase Health	0.503	0.485	1.077	1	0.299	1.654
Motivation: Time with Friends	Motivation: Increase Health	0.062	0.649	0.009	1	0.924	1.064
Motivation: Look Good	Motivation: Increase Health	0.086	0.338	0.064	1	0.8	1.089
**Registration Season: Spring**	**Registration Season: Summer**	**1.62**	**0.487**	**11.078**	**1**	**0.001**	**5.052**
Registration Season: Autumn	Registration Season: Summer	-0.174	0.543	0.103	1	0.749	0.84
**Registration Season: Winter**	**Registration Season: Summer**	**1.542**	**0.462**	**11.132**	**1**	**0.001**	**4.673**
Do not use a wearable	Use a wearable	-0.217	0.23	0.888	1	0.346	0.805
Use 7min Workout Regularly	Never use this app	-0.575	0.636	0.818	1	0.366	0.563
Use 7min Workout Sometimes	Never use this app	-0.119	0.287	0.171	1	0.679	0.888
Use 8Fit Planner Regularly	Never use this app	1.308	0.927	1.99	1	0.158	3.697
Use 8Fit Planner Sometimes	Never use this app	-0.345	0.513	0.451	1	0.502	0.709
Use Calm Meditation Regularly	Never use this app	-0.847	0.589	2.068	1	0.15	0.429
Use Calm Meditation Sometimes	Never use this app	0.47	0.311	2.288	1	0.13	1.6
Use Calorie Counter Regularly	Never use this app	0.33	0.539	0.375	1	0.54	1.391
Use Calorie Counter Sometimes	Never use this app	0.288	0.371	0.602	1	0.438	1.333
Use Fitbit Regularly	Never use this app	0.193	0.316	0.376	1	0.54	1.213
Use Fitbit Sometimes	Never use this app	0.255	0.355	0.517	1	0.472	1.291
Use Headspace Regularly	Never use this app	0.726	0.65	1.245	1	0.264	2.067
Use Headspace Sometimes	Never use this app	-0.099	0.332	0.089	1	0.766	0.906
Use MyFitnessPal Regularly	Never use this app	-0.059	0.307	0.038	1	0.846	0.942
Use MyFitnessPal Sometimes	Never use this app	0.099	0.258	0.147	1	0.702	1.104
Use Nike Regularly	Never use this app	0.176	0.451	0.153	1	0.696	1.193
Use Nike Sometimes	Never use this app	-0.368	0.298	1.525	1	0.217	0.692
Use Strava Regularly	Never use this app	0.62	0.375	2.733	1	0.098	1.859
Use Strave Sometimes	Never use this app	-0.286	0.371	0.593	1	0.441	0.752
Use WeightWatchers Regularly	Never use this app	-1.001	0.729	1.885	1	0.17	0.367
Use WeightWatchers Sometimes	Never use this app	0.603	0.529	1.3	1	0.254	1.828
